# Case of a Large Oropharyngeal Cyst

**DOI:** 10.7759/cureus.5843

**Published:** 2019-10-05

**Authors:** Joelle Brown, Matthew Carvey, Robert Hage

**Affiliations:** 1 Medicine, St. George's University School of Medicine, St. George's, GRD; 2 Otolaryngology, St. George's University School of Medicine, St. George's, GRD

**Keywords:** oral cyst, lymphoepithelial cyst, posterior tongue lesion

## Abstract

Non-odontogenetic oral cysts are rare occurrences in adults, especially when located in the oropharynx. We report a 35-year-old man with an oral cyst large enough to cause dysphagia of several years’ duration. The location of the swelling combined with the patient’s delay in seeking care and limited access to diagnostic tools prolonged the resolution of this case. Eventual puncture and marsupialization of the mass resulted in symptom relief. The patient remains cyst-free four years later.

## Introduction

Epithelial cysts are rare masses that can appear in a variety of organs, including the oral cavity [1]. Though they are typically benign, management of these cysts can be complicated by their location. A combination of clinical examination and computer tomography (CT) imaging may be necessary for effective diagnosis and treatment. Here we present a case of an unusually large cyst located in the oropharynx. The size and location of the cyst coupled with the patient’s tolerance of his symptoms for many years and limited access to diagnostic tools prolonged management of the condition. This case illustrates the importance of maintaining a broad differential diagnosis when evaluating oropharyngeal masses in the posterior area of the tongue, as lesions in this area are often unseen on initial examination.

## Case presentation

A 35-year-old man presented to a public clinic with recent speech distortion, causing a muffled voice. Past medical history was significant for 10 years of increasing difficulty with the initial phase of swallowing regardless of whether the bolus consisted of liquid or solid substances; however, the patient had no further issues with the remainder of the swallowing process. He did not feel as though something was lodged in his throat and reported no weight loss. Prior to this appointment, the patient had a barium swallow test that showed no abnormalities. He was a non-smoker and consumed alcohol occasionally. Past medical history was pertinent for hypertension controlled with 2.5 mg of Bezide daily and negative for acid reflux.

Physical examination was unremarkable: the tongue was mobile and the thyroid was not enlarged. No cervical lymphadenopathy was detected. The only tools available in the clinic for further investigation were a penlight and tongue spatula. Additional diagnostic modalities - specifically a flexible scope or a CT scan - were not offered at public clinics in this country and were accessible only to patients who could afford private health care. At this time, the patient did not have adequate funds for private care; therefore, further diagnostic procedures were put on hold.

**Figure 1:**
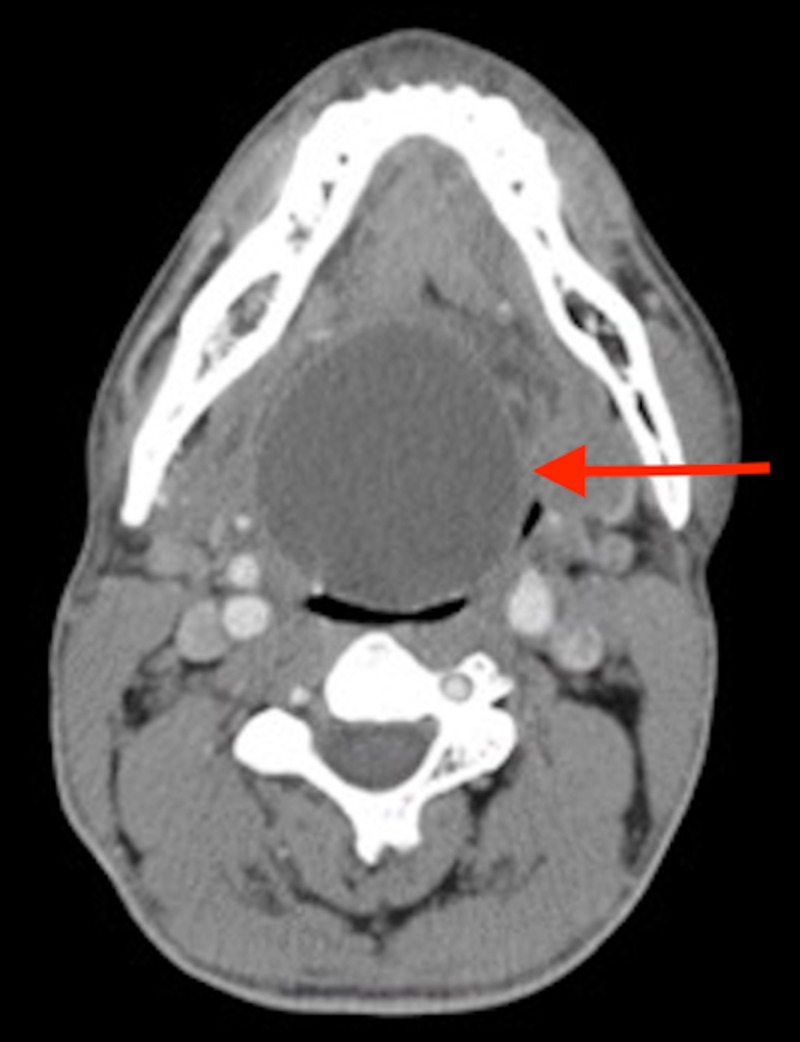
CT image of the mass lodged in the oropharynx, as indicated by the red arrow.

Two months after the initial visit, the patient was able to complete further testing in a private clinic. Flexible nasopharyngoscopy revealed a large, smooth mass off-midline, originating from the posterior one-third of the tongue and depressing the epiglottis. A subsequent CT scan (Figure 1) showed a large, midline, cystic space-occupying lesion in the suprahyoid neck, measuring 55x54x57 mm and inferior to the soft palate. No pooling of saliva or abnormalities of the arytenoids and posterior cricoid area were detected. Mass effect was noted on the posterior margin of the tongue, causing it to be displaced and buckled anteriorly. The epiglottis was displaced inferiorly. No calcifications, lymphadenopathy, or infiltration into adjoining soft tissue was seen. The thyroid gland was seen in its normal position with no abnormal communications. Retropharyngeal and parapharyngeal spaces were unremarkable, along with the laryngeal apparatus, salivary glands, carotid sheath, and strap muscles of the neck. The CT scan contradicted some of the findings of the flexible nasopharyngoscopy, specifically whether the mass occupied a midline or off-midline space in the oral cavity.

A subsequent examination under anesthesia (EUA) and biopsy were scheduled to explore the oropharynx. These services were performed in a public hospital free of charge. The EUA showed a large, smooth swelling, originating from the posterior tongue and obstructing the view of the laryngeal inlet. Given this location, the anesthetist was not comfortable proceeding with intubation. The surgeon decided to leave the mass intact and took only small tissue biopsies. Histological examination of one sample revealed a benign process consisting of stratified, squamous epithelium with vacuolization of cells and lymphoid infiltration. The lamina propria showed minor salivary glands and chronic inflammation. Examination of a second sample showed lymphoid infiltration with follicle formation, acanthosis, dyskeratosis, and severe dysplasia as indicated by irregular hyperchromatic nuclei and loss of nuclei to cytoplasm ratio. A biopsy in the submandibular triangle, performed to rule out a metastatic process, revealed normal submandibular gland tissue.

The patient was finally seen by an ear, nose, and throat (ENT) specialist, who managed to achieve oral intubation and aspirated 70 cc of fluid from the mass. The aspirate showed moderate cholesterol crystals in the fluid sediment and no cells or organisms. The surgeon incised the cyst, took a tissue sample, and marsupialized the cyst walls for continuous drainage. Gross imaging showed the cyst to be visually smooth; histological examination revealed thickened walls lined by flat epithelium with evidence of chronic inflammatory infiltrates.

The final diagnosis was a simple cyst on the posterior one-third of the tongue. Marsupialization proved an effective treatment procedure, as the patient made an uneventful recovery and remains symptom free four years later.

## Discussion

Oral cysts can be difficult to accurately diagnose. The unusually long history, large size, and oropharyngeal location of our patient’s cyst, coupled with a paucity of diagnostic tools, made this case particularly challenging. Differential diagnoses included thyroglossal duct cyst, salivary duct cyst, epidermoid cyst, and lymphoepithelial cyst.

Thyroglossal duct cysts (TGDC) are common congenital malformations of the neck related to the embryological development of the thyroid gland. The thyroid originates at the base of the tongue and migrates caudally to its permanent position in the inferior neck anterior to the trachea by the seventh week of gestation. The duct connecting the thyroid to the tongue typically atrophies by the tenth week of gestation; however, failure of this process can give rise to midline cysts that can be diagnosed at any age [2]. These cysts move with swallowing and are histologically characterized by respiratory epithelium, squamous epithelium, or a combination of the two. Lymphoid infiltration, as was seen in our patient, is possible but not typical [3]. Despite histological similarities with a TGDC, physical examination and imaging of our patient’s cyst did not suggest abnormal communication with the thyroid gland, making a TGDC unlikely.

Intraoral salivary duct cysts are acquired masses due to the obstruction of a major or minor salivary gland. They are typically characterized by columnar or cuboidal epithelium, though they may show squamous metaplasia [4]. Imaging in our patient did not show any obstructions or abnormalities of the salivary glands, making this diagnosis less plausible.

An epidermoid cyst is an encapsulated, subepidermal nodule that results from an obstructed follicular infundibulum. It has a stratified squamous epithelium and a keratin center in the subepidermal or dermal layers [5]. Though commonly found on the skin, there are case reports of epidermoid cysts in the buccal mucosa and in the oral cavity [6,7]. While histological examination of our patient’s cyst showed stratified, squamous epithelium, its lymphocytic contents ruled out an epidermoid cyst diagnosis.

Oral lymphoid cysts are rare, benign lesions that are generally considered branchial cleft derivatives due to their histological similarities [8]. They are typically found on the floor of the mouth or the ventral or lateral surfaces of the tongue [9]. There are two competing theories to explain their unusual development in the oral cavity. One supports a congenital explanation, in which cysts arise from epithelium trapped in the lymphoid aggregation of the oral cavity mucosa during embryogenesis [10]. The other asserts these cysts are part of an “oral tonsil” in which a crypt opening has become plugged, leading to enlargement [11]. These cysts usually consist of parakeratinized epithelium, though some had cuboidal, columnar, and/or squamous epithelium as well. They typically have lymphoid tissue surrounding the cystic lumen either partially or completely and are filled with desquamated keratinocytes, amorphous eosinophilic material, and/or inflammatory cells [1]. CT with contrast is the recommended initial diagnostic test; however, laryngoscopy can be used to achieve visualization if CT services are not available or unable to adequately characterize the mass [12]. Fine needle aspiration cytology (FNA) can then be performed to diagnose the mass in lieu of an open biopsy [13]. FNA should not be performed on pulsatile masses or masses of vascular origin. Management is usually surgical excision [12]. 

Of the four differential diagnoses, the description of the lymphoepithelial cyst most closely correlates with what was seen in our patient. However, the disease course for our patient was unorthodox. Other reports of similar cysts describe masses considerably smaller and in different regions of the oral cavity. For instance, in a study of 120 patients with oral lymphoepithelial cysts, 50% were found on the tongue and 38.3% on the floor of the mouth [14]. Another report from the Journal of Clinical Experimental Dentistry mentions 26 oral lymphoepithelial cysts, all less than 2 cm in size (range: of 0.3-1.7 cm), found on the ventral or lateral surface of the tongue (69.3%) or the floor of the mouth (30.7%) [1]. No patients in either report showed any sign of recurrence after surgical excision [1,14]. Thus, the large size and oropharyngeal location of our patient’s cyst makes his case unique.

## Conclusions

Oral cysts in adults are rare, benign masses that can prove difficult to diagnose, as they follow an indolent growth process and can be quite large before becoming symptomatic. Masses located in the posterior area of the tongue are difficult to visualize during a simple examination of the mouth and are often unseen on barium swallow tests, as was the case for our patient. Therefore, physicians must approach oropharyngeal lesions with a broad differential diagnosis in order to not overlook masses in this region and unintentionally prolong the impact these lesions have on a patient’s quality of life.
